# Surgical outcomes of indocyanine green fluorescent cholangiography in emergency laparoscopic cholecystectomy performed by junior surgeons for acute cholecystitis

**DOI:** 10.3389/fsurg.2026.1881022

**Published:** 2026-07-09

**Authors:** Lei Gao, Jun He, Yifeng Qian, Fei Yuan, Wei Liu, Yanping Lu, Gang Qian, Yefei Mao

**Affiliations:** 1 Department of General Surgery, Zhangjiagang Third People’s Hospital, Zhangjiagang, China; 2 Department of Anesthesiology, Zhangjiagang Third People’s Hospital, Zhangjiagang, China

**Keywords:** acute cholecystitis, emergency, indocyanine green, junior surgeon, laparoscopic cholecystectomy

## Abstract

**Background:**

Emergency laparoscopic cholecystectomy (LC) remains the preferred treatment for acute cholecystitis (AC); however, the prevalence of complications and negative outcomes increases in these emergency surgeries, especially when performed by junior surgeons. Recently, indocyanine green (ICG) fluorescent cholangiography has emerged as a useful technique for achieving adequate visualization of the biliary structures during LC, reducing the incidence of bile duct injury (BDI) and operative time. This study aims to evaluate the surgical outcomes of ICG fluorescent cholangiography during emergency LC performed by junior surgeons.

**Methods:**

A total of 156 consecutive patients (May 2023  to  December 2025) who underwent LC for AC were included, and all the surgeries were performed by junior surgeons. In total, 78 patients underwent ICG fluorescent cholangiography during LC (ICG group), while the remaining 78 patients underwent conventional LC with white light (non-ICG group). The demographic and clinical characteristics of these patients were retrospectively collected and analyzed, including gender, age, comorbidities, intraoperative BDI, conversion to open surgery, operative time, postoperative complications, postoperative hospital stay duration, and overall hospitalization cost. A multiple linear regression analysis was performed to examine whether ICG use was associated with the surgical outcomes.

**Results:**

The demographic characteristics of the ICG and non-ICG groups were similar. The patients in the ICG group had a significantly shorter operative time compared to those in the non-ICG group (65 min vs. 72.5 min, *P*  =  0.007). There were no significant differences between the groups for the other surgical outcomes, including BDI incidence, rate of conversion to open surgery, incidence of postoperative complications, postoperative hospital stay duration, and hospitalization cost. ICG use was found to be an independent factor associated with decreased operative time in the regression analysis.

**Conclusions:**

The utilization of ICG fluorescent cholangiography during emergency LC could help junior surgeons shorten the operative time and may not increase the incidence of perioperative complications.

## Introduction

Acute cholecystitis (AC) is one of the most common diseases requiring emergency surgery. Gallstone-associated cystic duct obstruction is responsible for 90%–95% of AC cases, and laparoscopic cholecystectomy (LC), performed within 3 days of diagnosis, is the first-line therapy for AC (1). LC performed within 1–3 days of symptom onset is associated with improved patient outcomes, including fewer composite postoperative complications, a shorter hospital stay duration, and lower hospital costs (1, 2). The Tokyo Guidelines 2018 (TG18) recommends LC within 24–72 h of symptom onset, while the 2020 World Society of Emergency Surgery (WSES) recommends LC within 7 days of hospital admission and within 10 days of symptom onset (3, 4).

Although generally considered relatively safe, the LC procedure has an approximate mortality rate of 0.3%–0.5% (5). The most serious complication of LC is bile duct injury (BDI), resulting in a significant impact on quality of life, overall survival, and medico-legal liabilities (6). The annual incidence of iatrogenic BDI after LC has not declined significantly in recent years (7). The primary cause of BDI is misinterpretation of the biliary anatomy, occurring in 71%–97% of all cases (8). Certain factors may increase the incidence of BDI. For example, AC causes a series of modifications of the local anatomy (adhesions, thickening of the tissues, inflammation, and bleeding) that are associated with an increased risk of iatrogenic lesions (6). The surgeon's level of experience is associated with adverse outcomes in LC, with 90% of BDIs occurring in surgeons’ first 30 cases (9).

Indocyanine green (ICG) fluorescence is a useful strategy for enhancing the visualization of anatomical structures in real time. In recent years, it has emerged as one of the most promising and rapidly developing technical innovations in surgery. One of its most popular current clinical applications is fluorescent cholangiography (10). ICG is a water-soluble dye that has low reported rates of adverse events and allergic reactions. After hepatic extraction, the dye is almost entirely eliminated in the bile after binding with circulating albumins and lipoproteins. Its half-life in the blood is 3–5 min (11). Both intravenous injection and direct injection into the gallbladder can be useful for identifying the extrahepatic biliary anatomy (12, 13). The WSES consensus recommends the use of ICG cholangiography during LC for severe cholecystitis, as it allows the correct and real-time visualization of the extra-hepatic biliary tree anatomy. Moreover, ICG cholangiography reduces the rates of BDI and conversion to open surgery in the emergency setting in selected patients (14). Although ICG is recommended in emergency LC, its benefits for junior surgeons lacking surgical experience are still unvalidated (15–17).

This study aims to evaluate the impact of ICG fluorescent cholangiography during emergency LC for AC performed by junior surgeons by comparing the surgical outcomes, including incidence of BDI and operative time, with conventional (white light alone) LC.

## Methods

### Study design and patients

This retrospective study was conducted from May 2023 to December 2025 at the General Surgery Department of Zhangjiagang Third People's Hospital. A consecutive series of patients who underwent emergency LC performed by junior surgeons within 24 h of admission was enrolled in this study.

The surgeries were performed by four different junior surgeons with at least 2 years of surgical experience each. These surgeons had passed certified laparoscopic technique training courses before performing LC as the chief surgeon. All of the junior surgeons had performed at least 10 elective LC procedures as the chief surgeon and had no experience performing LC as the chief surgeon in an emergency setting. Only the initial 40 emergency LC cases performed by these junior surgeons were included in this study. The patients were sorted by surgery date, ranging from 1 to 160 in total. Patients assigned odd numbers were placed in the ICG group, while those patients assigned even numbers were placed in the non-ICG group.

All patients were diagnosed with AC based on the TG18 diagnostic criteria, which were as follows: (a) local signs of inflammation (Murphy's sign or right upper quadrant mass/pain/tenderness), (b) systemic signs of inflammation (fever, elevated C-reactive protein, or elevated white blood cell count), and (c) imaging findings characteristic of AC (18). This study was approved by the Ethics Committee of Zhangjiagang Third People's Hospital (ZJGSY-2023-04) and was conducted in accordance with the Declaration of Helsinki. All the patients provided written informed consent.

Patients aged from 18 to 80 years old who underwent LC performed by junior surgeons were included. The exclusion criteria were as follows: patients with an allergy to ICG or iodine, those with suspected gallbladder malignancy preoperatively, those with severe cardio-respiratory diseases that were contraindicated for laparoscopic surgery, those with a preoperative diagnosis of cholangitis and pancreatitis, and those who underwent LC and another surgical procedure simultaneously, such as common bile duct (CBD) surgery.

### Surgical procedure

LC was performed using the conventional three-port approach, with a 10-mm umbilical trocar for laparoscopic observation and a 10-mm xiphoid trocar and a 5-mm trocar in the right subcostal region for the surgical instruments. An additional 5-mm trocar may be placed in the right lower abdomen area for assistance if necessary. Intra-abdominal pressure was kept at 12–14 mmHg. Gallbladder decompression was applied if the gallbladder was too tense and distended to be grasped and to explore the surgical field. Dissection of Calot's triangle using a monopolar hook and suction irrigator was performed until the critical view of safety (CVS) was achieved, if possible. Once the cystic duct and gallbladder artery were identified, they were clipped and cut. The gallbladder was dissected and then extracted through the umbilical trocar. When the inflammation and adhesion in Calot's triangle were too severe to distinguish the biliary structures, such as the cystic and common hepatic ducts, the fundus-first approach was used to complete the procedure. Several alternative safety strategies may be conducted to prevent BDI, including subtotal cholecystectomy, bailout strategies, and conversion to open surgery. Hemostasis was confirmed and irrigation of the surgical field was conducted. As all the surgeries were performed by junior surgeons, a drainage tube was placed to evaluate postoperative bleeding and bile leakage. The drainage tube was removed on the second day after surgery if there was no evidence of bleeding or bile leakage. Senior surgeons mainly provided assistance and supervision during the surgeries, and they did not take over procedures unless a BDI or massive bleeding occurred that the junior surgeons could not deal with.

**Figure 1:**
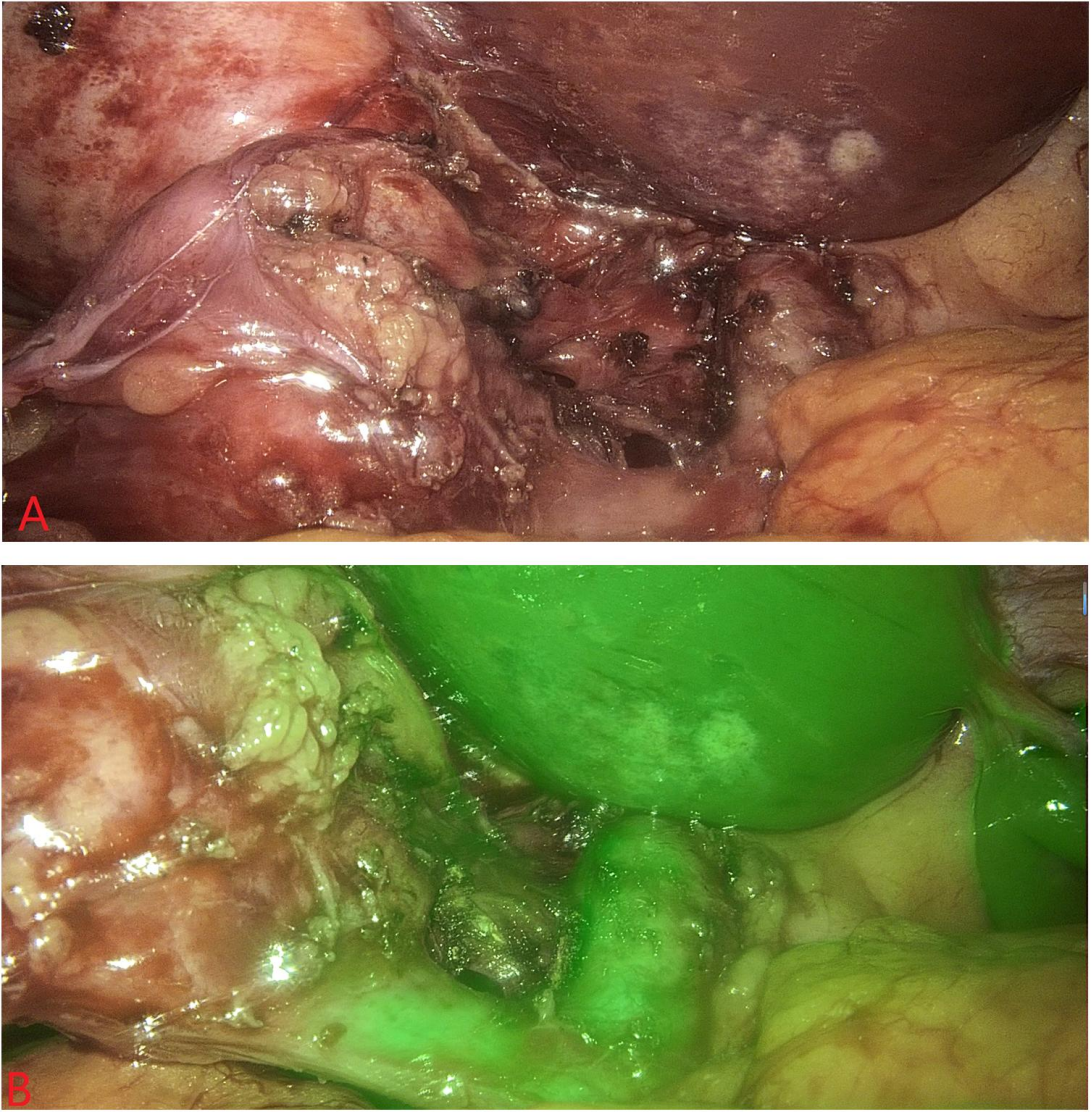
Biliary anatomy visualization in acute cholecystitis with white light **(A)** and with indocyanine green **(B)**

For patients who underwent intraoperative ICG fluorescent cholangiography, 2.5 mg of ICG (manufactured by Dandong Yichuang Pharmaceutical Co., LTD, China) was administered via peripheral intravenous injection 30–60 min before anesthesia. The near-infrared ICG fluorescent imaging system used in our center was the 4K Fluorescence Endoscopic System (Zhejiang Healnoc Technology Co., Ltd, China). Visualization was in the white light mode for the majority of the procedure, with ICG fluorescent cholangiography mainly performed during the exposure and dissection of Calot's triangle to achieve CVS. After the verification of the cystic duct and gallbladder artery, the imaging system switched back to white light. Before removing the surgical instruments and closing the incisions, the imaging system switched back to ICG for a final observation of the surgical field. Biliary anatomy visualizations during emergency LC using white light and ICG are shown in Figures 1A,B.

### Outcomes

The baseline demographics and characteristics of the patients were retrospectively collected, including gender, age, body mass index (BMI), American Society of Anesthesiologists (ASA) classification, comorbidities, abdominal surgery history, duration from symptom onset to surgery, severity of cholecystitis based on the TG18 grade, selected inflammatory markers on the first day of admission, and gallbladder wall thickness measured under ultrasound or CT scan.

The primary outcomes were intraoperative complications, including massive blood loss, BDI occurrence, and conversion to open surgery. The operative time was also recorded to evaluate the efficiency of the ICG fluorescent cholangiography. The secondary outcomes included postoperative complications, postoperative hospital stay duration, overall hospitalization cost, readmission, and reoperation. The follow-up time was within 30 days after LC. Data on the contents and pathology of the gallbladder were also collected. All surgical outcome data were retrospectively collected.

### Statistical analysis

The statistical analysis was performed using SPSS statistical software (version 25.0, IBM Corporation, New York, USA). A sample size estimation was not conducted due to the exploratory nature of this retrospective study, which comprised surgeries performed by junior surgeons at our center. The Kolmogorov–Smirnov test was used to assess data normality. The normally distributed continuous numerical variables are expressed as mean [standard deviation (SD)] and were analyzed using the independent sample *t*-test. The non-normally distributed continuous numerical variables are expressed as median [interquartile range (IQR)] and were analyzed using the Mann–Whitney *U* test. The categorical variables are represented as counts and were analyzed using the chi-squared or Fisher's exact test. A multiple linear regression analysis was performed to examine whether ICG use was independently associated with the surgical outcomes. A *P*-value <0.05 indicated a statistically significant difference.

## Results

A total of 156 patients were included in this study. Three surgeons performed 40 surgeries, while one surgeon performed 36 surgeries during the study period. In total, 78 patients who underwent intraoperative ICG fluorescent cholangiography were included in the ICG group, while the remaining 78 patients who underwent the conventional (white light) procedure were included in the non-ICG group.

The baseline demographics and characteristics of the patients before surgery are shown in Table 1. There were 41 male patients and 37 female patients in the non-ICG group, while there were 38 male patients and 40 female patients in the ICG group (*P* = 0.631). There were no statistically significant differences between the two groups in terms of age (*P* = 0.976), BMI (*P* = 0.935), ASA classification (*P* = 0.591), comorbidities (*P* > 0.05), abdominal surgery history (*P* = 0.547), history of cholecystitis attack (*P* = 0.337), and gallbladder wall thickness (*P* = 0.233). The median (IQR) duration from symptom onset to surgery in the non-ICG group was 2 (1–4) and 2 (2–5) days in the ICG group (*P* = 0.671). In both groups, the severity of cholecystitis according to TG18 was predominantly grade II, followed by grade I, and only one patient in the non-ICG group was diagnosed with severity of grade III (*P* = 0.225). There were no significant differences between the two groups in inflammatory markers, including white blood cell count, neutrophil percentage, C-reactive protein, and procalcitonin, on the first day of admission (*P* > 0.05).

**Table 1:** Preoperative demographic and clinical characteristics of the patients.

Variable	Non-ICG group (*n *= 78)	ICG group (*n *= 78)	*P*-value
Gender (male/female)	41/37	38/40	0.631
Age (year)	55.91 (13.64)	55.97 (13.40)	0.976
Body mass index (kg/m^2^)	23.99 (2.05)	23.96 (2.62)	0.935
ASA			0.591
I	58	55	
II	20	23	
Comorbidities			
Hypertension	25	29	0.501
Diabetes	9	9	>0.999
Cardiac heart disease	1	5	0.210
Cerebral infarction	3	1	0.620
Chronic renal failure	2	0	0.497
HBV hepatitis	1	0	>0.999
Gouty arthritis	1	1	>0.999
Ankylosing spondylitis	0	1	>0.999
Hypothyroidism	1	0	>0.999
Anticoagulation therapy	4	3	>0.999
Abdominal surgery history	17	14	0.547
History of cholecystitis attack	42	36	0.337
Duration before surgery (day)	2 (1,4)	2 (2,5)	0.671
Severity grade (Tokyo Guidelines 2018)			0.225
I	31	23	
II	46	55	
III	1	0	
White blood cell count (*10^9^/L)	8.83 (5.99,11.5)	8.52 (7,12)	0.304
Neutrophil percentage (%)	75.6 (64.4,84.8)	80.1 (70,88.2)	0.059
C-reactive protein (mg/L)	6.91 (3.12,13.85)	4.74 (2.44,23.48)	0.727
Procalcitonin (ng/mL)	0.05 (0.03,0.09)	0.05 (0.03,0.08)	0.844
Gallbladder wall thickness (mm)	3 (2,5)	3(2,4)	0.233

Values are presented as mean (SD), median (IQR), or number.

ICG, indocyanine green; ASA, American Society of Anesthesiologists; HBV, hepatitis B virus.

As shown in Table 2, the median operative time was shorter in the ICG group than in the non-ICG group, with a statistically significant difference (65 min vs. 72.5 min, *P* = 0.007). In both groups, at least 57% of the surgeries were completed within 60–90 min, and the number of surgeries lasting more than 90 min was lower in the ICG group than in the non-ICG group (*P* = 0.078). Regarding the primary outcomes, there were no significant differences in blood loss, BDI occurrence, and conversion to open surgery between the two groups (*P* > 0.05). One patient in the non-ICG group experienced a complete transection of the CBD and a senior surgeon performed a Roux-en-Y hepaticojejunostomy with an open approach. One patient in the ICG group had bile leakage from a small duct in the liver bed after the gallbladder was dissected. The injured duct was immediately sutured using a 3-0 absorbable suture. No statistically significant differences were found between the two groups regarding postoperative complications (*P* = 0.632), postoperative hospital stay duration (*P* = 0.369), and hospitalization cost (*P* = 0.424). Moreover, there were no significant differences in gallbladder content (*P* = 0.209) and pathological ﬁnding (*P* = 0.759) between the two groups. No readmissions or reoperations occurred during the follow-up time.

**Table 2:** Surgical outcomes of the patients.

Variable	Non-ICG group (*n *= 78)	ICG group (*n *= 78)	*P*-value
Operative time (min)			0.078
<60	13	23	
60–90	47	45	
>90	18	10	
Operative time (min), median	65 (55,75)	72.5 (60,90)	0.007
Blood loss (mL)			0.753
<20	65	66	
20–50	8	9	
>50	5	3	
Blood loss (mL), median	5 (5,10)	5 (5,10)	0.889
Intraoperative bile duct injury	1	1	>0.999
Conversion to open surgery	1	0	>0.999
Postoperative complications (total)	11	9	0.632
Clavien–Dindo classification I	4	4	
Increased liver enzymes	0	1	
Diarrhea	1	1	
Surgical site infection	1	1	
Peritoneal fluid accumulation	2	1	
Clavien–Dindo classification II	7	5	
Pneumonia	3	1	
Deep vein thrombosis	0	1	
Arrhythmia	1	0	
Cholangitis	0	2	
Hydronephrosis with renal insufficiency	1	0	
Paralytic ileus	1	0	
Biliary-intestinal anastomotic leakage	1	0	
Acute cerebral infarction	0	1	
Gallbladder content			0.209
No gallbladder stones or polyps	2	3	
Concomitant gallbladder stones	72	65	
Concomitant gallbladder stones and polyps	4	10	
Pathology			0.759
Acute cholecystitis	51	55	
Pyogenic cholecystitis	14	11	
Gangrenous cholecystitis	13	12	
Postoperative hospital stay duration (day)	4 (3,5)	4 (3,5)	0.369
Hospitalization cost (RMB, Yuan)	9900 (9076,10998)	10051 (9357,11140)	0.424
Readmission	0	0	N/A
Reoperation	0	0	N/A

Values are presented as median (IQR) or number.

ICG, indocyanine green.

The result of the multiple linear regression analysis with operative time as the outcome is presented in Table 3. The use of ICG was found to be an independent factor associated with decreased operative time in the regression analysis.

**Table 3:** Multiple linear regression analysis with operative time as the outcome.

Variable	Unstandardized B	*P*-value	95% CI
Constant	72.286	0.007	20.462 to 124.111
Gender (male/female)	6.902	0.121	−1.836 to 15.640
Age (year)	−0.133	0.454	−0.482 to 0.217
Body mass index (kg/m^2^)	−0.721	0.429	−2.518 to 1.075
ASA (I/II)	−6.828	0.169	−16.587 to 2.931
History of cholecystitis attack	−2.253	0.594	−10.586 to 6.079
Duration before surgery (day)	−1.099	0.248	−2.971 to 0.774
Severity grade (Tokyo Guidelines 2018) (I/II/III)	8.019	0.106	−1.725 to 17.763
White blood cell count (*10^9^/L)	0.430	0.529	−0.918 to 1.778
Neutrophil percentage (%)	0.119	0.533	−0.256 to 0.493
C-reactive protein (mg/L)	0.008	0.895	−0.108 to 0.124
Procalcitonin (ng/mL)	−0.140	0.955	−5.059 to 4.778
Gallbladder wall thickness (mm)	2.173	0.078	−0.245 to 4.592
Pathology (acute cholecystitis/pyogenic cholecystitis/gangrenous cholecystitis	4.520	0.207	−2.524 to 11.565
ICG use	−11.216	0.007	−19.281 to −3.151

ASA, American Society of Anesthesiologists; CI, confidence interval; ICG, indocyanine green.

## Discussion

When performing LC, junior surgeons with less experience find it difficult to distinguish between the biliary structures, especially in emergency cases complicated by inflammation or scarring, leading to adverse surgical outcomes. Previous studies have reported that the application of ICG fluorescent cholangiography was beneficial in elective LC (19–21). However, there was limited evidence regarding the usefulness of ICG in emergency settings and in surgeries performed by junior surgeons. In our retrospective study, the baseline clinical variables of the two groups showed no significant differences. Therefore, the analysis minimized the influence of potential selection bias and the two surgical approaches were comparable. We demonstrated that ICG fluorescent cholangiography may help junior surgeons reduce operative time in emergency LC.

BDI is the primary concern for all surgeons during LC. Several techniques have been proposed to prevent BDI, with the most critical being the establishment of a CVS (22). However, in AC cases, achieving the correct CVS may not always be possible or safe. Several safety strategies have been reported to minimize the risk of BDI during LC. These strategies include an intraoperative cholangiogram, bailout procedures, the fundus-first approach, laparoscopic subtotal cholecystectomy, and conversion to open surgery. Moreover, the operating surgeon should pause and seek a second opinion from another surgeon during a difficult LC (22). As we compared surgical outcomes between ICG fluorescence and the white light mode, intraoperative cholangiograms were not performed in our study. The fundus-first approach was applied during procedures to prevent BDI in our center. However, we consider the use of the fundus-first approach to be related to severe inflammation or biliary inflammatory adhesions and scarring in Calot's triangle, and an analysis of its correlation with ICG was not performed.

In a multicenter randomized controlled trial, van den Bos et al. concluded that the use of ICG in LC provided earlier identification of relevant extrahepatic biliary anatomy, leading to earlier achievement of CVS, cystic duct visualization, and visualization of both the cystic duct and the cystic artery transition into the gallbladder (23). An earlier meta-analysis including 3,457 patients showed that ICG fluorescent cholangiography was beneficial for early real-time visualization of the biliary structure, shorter operative time, and lower risk of conversion during LC (24). Another meta-analysis published in 2025 reported decreased operative time when using the ICG fluorescent cholangiography technique in difficult LC (25). Our study had a similar finding, as operative time in the ICG group was shorter than that in the non-ICG group. However, Ramírez-Giraldo et al. did not observe a reduced operative time when using ICG fluorescent cholangiography in AC (16). Approximately 80% of the surgeries in Ramírez-Giraldo et al.'s study were performed by senior surgeons, and their surgical proficiency may mitigate the advantages of ICG fluorescent cholangiography.

A longer operative time in LC was positively correlated with the incidence of perioperative complications (26). Another study on general surgery showed that operative time was independently associated with increased complication rates and length of stay (27). Therefore, shortening the operative time is significant for perioperative management. In our multiple linear regression analysis, the utilization of ICG was an independent factor associated with decreased operative time. Local adhesions and inflammation in Calot's triangle are common and severe in AC cases, increasing the difficulty of emergency LC. For inexperienced junior surgeons, dealing with these issues is challenging, and the operative time can be significantly prolonged. However, through the use of ICG, the operative time can be shortened.

The incidence rate of BDI has been reported to be approximately 0.2%–1.5% (28, 29). In our study, one patient in each of the two groups had a BDI and the incidence rate was 1.3%, which was consistent with previous studies. Several risk factors contribute to the incidence of iatrogenic BDI, namely, anatomical factors, patient-related factors, emergency surgery, acute or subacute cholecystitis, the surgical technique used, and the surgeon’s ability (6, 29, 30). The surgeon's inability to identify normal anatomy inside Calot's triangle is the predominant cause of iatrogenic BDI (30). The CBD injury in our study was due to the junior surgeon's misidentification of the biliary structures inside Calot's triangle when using the conventional white light method. This BDI is classified as a type E1 injury in the Strasberg classification (31). Although there was one case of BDI in the ICG group, the injured duct was a small duct in the liver bed (type A injury in the Strasberg classification), and no important biliary structures, such as the CBD, common hepatic duct, and right hepatic duct, were injured when using the ICG method. The small duct injury was related to the junior surgeon's technique when dissecting the gallbladder. Thus, the injury that occurred in the non-ICG group was far more severe than that in the ICG group. However, due to the insufficient sample size, a connection between BDI incidence and ICG use could not be confirmed in our study. Liu et al. reported that ICG cholangiography helped young surgeons successfully perform 97 cases of emergency LC without any intraoperative iatrogenic injuries (15). In Haverinen et al.'s study, including 2,009 patients, the incidence of BDI was lower with ICG fluorescence than without ICG in LC using the fundus-first technique (0.4% vs. 0.5%, *P* = 0.69) (32). Due to the low incidence of BDI, it is challenging to detect statistical significance as sample sizes are small. However, even if the incidence of BDI is reduced by one case, patients’ quality of life will improve and medical disputes can be avoided.

No significant differences were observed in terms of conversion rate, postoperative hospitalization stay duration, cost, and overall postoperative complications in our study. A randomized controlled trial, including 92 patients, explored the effectiveness of ICG fluorescence cholangiography in emergency LC, finding that it led to no difference in the conversion or complication rate (33). Ramírez-Giraldo et al. reported that the length of stay and major complication rate were similar in conventional and fluorescence-guided cholecystectomy (16). In Losurdo et al.'s study, which focused on emergency settings, the use of ICG reduced complications and length of hospital stay, and the conversion rate was lower than 4%, which was inferior to the reported range in a previous study (34). A recent study including 87 patients who underwent emergency surgery for AC found that ICG may help reduce the need for conversion to open surgery and median hospital stay. However, the use of ICG has not been proven to reduce bile duct injury, postoperative complications, or operative time (35). Although the above studies differed slightly from the conclusions of our research, the use of the ICG technology did not increase the conversion rate or number of postoperative complications as a whole. We believe that the sample size of the study and the experience of the surgeons may have had an impact on the results. In addition, due to the lack of consensus on the use of ICG, including the dose, the timing of administration, and the injection approach, the use of ICG fluorescent cholangiography in different studies was not completely consistent, which may also lead to bias between different studies. A recent meta-analysis demonstrated that lower liver fluorescence with an intrabiliary ICG injection may enhance visual clarity, while operative time was slightly longer compared with the intravenous ICG injection approach (36).

This study had several limitations. First, this was a single-center retrospective study with a small sample size and only the initial 40 cases of emergency LC performed by four junior surgeons were included, which introduced some bias. This study may not be sufficiently powered to elucidate the impact of ICG use on BDI incidence or other postoperative complications. Randomized controlled trials with a large sample size are required in future studies. Second, the usage of ICG in our center was based on our previous clinical experience. The optimal dosage and timing of ICG administration in emergency settings remain unknown and need to be validated in the future. Third, we failed to assess the learning process of the junior surgeons, as all the junior surgeons had experienced several elective procedures of LC before participating in this study. Moreover, all the junior surgeons used ICG in half of their initial emergency LC procedures, which posed a significant obstacle to depicting their learning process.

## Conclusions

In conclusion, ICG fluorescent cholangiography is effective and safe in emergency LC performed by junior surgeons for AC. The utilization of ICG fluorescent cholangiography can shorten the operative time. Further randomized controlled trials with a large sample size are warranted to validate these findings.

## Data Availability

The raw data supporting the conclusions of this article will be made available by the authors, without undue reservation.
